# Forensic DNA elimination databases in Europe: A comparative analysis of data from seven countries

**DOI:** 10.1016/j.fsisyn.2025.100617

**Published:** 2025-06-19

**Authors:** Mónika Nogel, Zsolt Pádár, Gábor Kovács

**Affiliations:** a Széchenyi István University, Faculty of Law and Political Sciences, Department of Criminology and Forensic Sciences, Hungary; b Ludovika University of Public Services, Institute of Information Society, Hungary

**Keywords:** Forensic DNA elimination databases, DNA contamination management, Forensic DNA database

## Abstract

Forensic DNA elimination databases are valuable tools for identifying potential contamination risks during forensic investigations. This study provides a comparative analysis of the design, implementation, and effectiveness of forensic DNA elimination databases across seven European countries: Sweden, Germany, Czechia, Poland, the Netherlands, the UK, and Finland. Data were collected through structured inquiries sent to ENFSI member states’ forensic DNA laboratories, focusing on key aspects such as legal frameworks, database sizes, and contamination cases identified through the elimination database. The results reveal significant differences in the establishment and management of these databases, reflecting diverse legal and operational contexts. The findings underscore the need to ensure that all European countries implement their elimination databases to standardize forensic procedures and improve the reliability of DNA evidence. Furthermore, the study highlights the importance of introducing harmonized frameworks for forensic DNA elimination databases to enhance transparency, accessibility, and efficiency in forensic DNA practices across Europe.

## Highlights

• A comparative analysis of forensic DNA elimination databases was conducted in seven EU countries.
• Significant variability was observed in database sizes, legal frameworks, and operational practices.
• Robust practices in Czechia, Poland, and Sweden could serve as potential models for other nations.
• Highlights the need for harmonized European frameworks for database management.

## 1 Introduction

Over recent years, advancements in genetic technologies have dramatically enhanced the sensitivity and analytical capabilities of forensic DNA analysis [[1], [2], [3], [4]].

These improvements have made it possible to generate genetic profiles from even the smallest quantities of biological material [5]. This progress has enabled more precise matches and the resolution of complex cases that would have been unsolvable in the past, revolutionizing forensic investigations by significantly contributing to the growing success of forensic DNA databases worldwide [[6], [7], [8], [9]]. However, the increased sensitivity of these methods has also introduced new challenges, particularly the heightened risk of detecting genetic profiles unrelated to the case [[10], [11], [12], [13], [14], [15]].

Profiles mistakenly believed to belong to a perpetrator may, in reality, originate from external sources, such as crime scene personnel, first responders (e.g., medical staff), or other individuals who inadvertently introduce contamination [16,17,17,18,18,19,19,20].

Contamination poses significant risks to forensic investigations [20]. Misleading genetic evidence can divert investigative efforts, consume resources, and prolong the resolution of cases in general [17,21]. As a result, managing and mitigating these risks has become an essential priority in forensic science, requiring robust preventative strategies to ensure the reliability of DNA evidence [[22], [23], [24],[24], [25], [26], [27]]. To mitigate such risks, forensic laboratories have implemented strict protocols, including using sterile, single-use equipment, maintaining proper chain-of-custody procedures, and ensuring separation of pre- and post-PCR processes. Robust quality management systems also play a critical role in this effort. Adhering to international standards, such as ISO/IEC 17025 for testing and calibration laboratories, ensures that every aspect of forensic analysis—from sample collection to final reporting—is carried out with precision, consistency, and accountability. Regular audits, proficiency testing, and validation of methods are essential components of these systems, helping to identify potential issues before they compromise investigations [21,[25], [26], [27], [28], [29], [30], [31]].

One of the proactive measures increasingly adopted across Europe is the implementation of forensic DNA elimination databases. A DNA elimination database specifically helps detect contamination that might occur during the DNA analysis process. It helps save resources, prevents police from following false leads, and reduces delays in court cases. These databases usually include the DNA profiles of authorized personnel, like crime scene investigators and forensic analysts, who may come into contact with evidence. By comparing unknown DNA to these profiles, investigators can quickly spot and rule out potential contamination [32].

In Europe, the European Network of Forensic Science Institutes (ENFSI) has played a pivotal role in promoting the adoption of forensic DNA elimination databases. The ENFSI DNA Working Group first recommended the establishment of such databases in their *DNA Database Management Review and Recommendations* in 2009. These recommendations were reinforced in subsequent years, emphasizing the importance of accompanying every forensic DNA database with an elimination DNA database. ENFSI recommended including DNA profiles of individuals who might have inadvertently contaminated evidence, such as lab personnel, cleaning staff, investigating officers, and others present at crime scenes. Furthermore, unidentified DNA profiles from negative controls, which may have originated from individuals involved in manufacturing disposables or chemicals, were recommended for inclusion and sharing among ENFSI member countries. However, while these recommendations highlighted best practices, their implementation varied, and not all countries or laboratories introduced such databases [26].

In countries with elimination databases, their purpose is the same. When a match is found between a crime scene profile and one in the elimination database, it helps identify contamination. Detecting contamination enables investigations to confirm its source and supports the implementation of proactive measures and training to prevent future occurrences [27,[33], [34], [35]].

This paper presents a comparative analysis of forensic DNA elimination databases in seven European countries that are members of the ENFSI, including Sweden, Germany, Czechia, Poland, the Netherlands, the UK, and Finland. Through this analysis, the study aims to support the development of standardized best practices for managing forensic DNA evidence across Europe.

## 2 Methods

Between January and September 2024, structured data collection targeted forensic DNA laboratories within the ENFSI member institutions located in European countries. The publicly available contact details for these laboratories were obtained from the ENFSI website (https://enfsi.eu/about-enfsi/members/).

A standardized email was sent to the identified institutions, requesting specific information about the establishment, legal framework, size, and operational practices of forensic DNA elimination databases in their respective countries. The email included clearly defined questions designed to elicit detailed and structured responses.

• **Existence of a forensic DNA elimination database**: Institutions were asked to confirm whether such a database had been established and, if applicable, provide an overview of its scope (e.g., personnel covered, such as forensic staff, law enforcement officers, or visitors).
• **Date of introduction and legal framework**: Respondents were asked to indicate the year the database was introduced and provide details of the relevant legal or regulatory background.
• **Size of the database**: Institutions were asked to report the total number of elimination DNA samples currently recorded, specifying the date of the provided data.
• **Annual breakdown of DNA samples submitted:** Respondents were requested to provide a detailed year-by-year breakdown of DNA sample submissions since the database's establishment.
• **Database matches with crime scene samples:** Respondents were requested to provide annual data on the number of matches between crime scene DNA profiles and elimination database entries.

Responses were received in narrative format and subsequently standardized for analysis during the research period. Where additional clarification was required, follow-up emails were sent to respondents. All collected data were stored securely and analyzed to identify trends, variations in implementation, and the effectiveness of forensic DNA elimination databases across different jurisdictions.

## 3 Results

Out of the forensic DNA laboratories operating in ENFSI member states located in Europe, seven laboratories provided detailed data regarding their forensic DNA elimination databases, including Sweden, Germany, Czechia, Poland, the Netherlands, the UK and Finland. The responses varied significantly in scope and content, reflecting the diversity in practices and regulations. The overall response rate highlights both the opportunities and challenges in obtaining data on this sensitive topic.

Several laboratories confirmed that no forensic DNA elimination database existed in their country at the time of the request, including Moldova and Norway.

One laboratory (France) reported having an elimination database but was unable to share data due to legal restrictions. Another laboratory (Hungary) confirmed the existence of a database but declined to provide data without offering further justification.

A subset of laboratories did not respond to the inquiry, despite multiple follow-up attempts. This highlights the varying approaches and attitudes toward sharing data for research purposes.

Laboratories that provided detailed responses offered insights into the implementation, management, and usage of their forensic DNA elimination databases. Table 1 summarizes key data points, including the year of establishment, the number of samples in the database, and the regulatory framework governing its operation. The data underlines the complexity of the subject and the influence of national regulatory and operational frameworks.

**Table 1:** Overview of forensic DNA elimination database practices in some European countries.

***Country***	***Database established***	***Legal basis***	***Samples in database****(as of 2024)*	***Annual breakdown***	***Contamination cases recorded****(Total)*	***Contamination cases recorded****(Annually)*	***Source of data in the database***
*Czechia*	2008 (expanded in 2011, regulated in 2016)	Czech Police President's Guideline 275/2016 (legally binding)^a^	∼3900	until 2018: 2619	1235	Until 2018: 531	Police officers, forensic technicians, and laboratory staff; mandatory inclusion for regulated groups
				2018: 320		2018: 36	
				2019: 253		2019: 117	
				2020: 18		2020: 121	
				2021: 176		2021: 119	
				2022: 899		2022: 132	
				2023: 320		2023: 168	
				2024: no data available		2024: no data available	
*Poland*	September 2020	Polish Police Act (Journal of Laws 1990 No. 30, item 179), Article 20 (1l), Articles 21a-e, Regulation of the Minister of Internal Affairs (Journal of Laws 2020, item 1347)^b^	9028	2020: 63	403	2020: 1	Police officers and employees of criminal services
				2021: 5741		2021:272	
				2022: 1101		2022: 49	
				2023: 1740		2023: 81	
				2024^c^: 383		2024: 0	
*Sweden*	July 2014	Swedish Law 2014:400 on Forensic DNA Elimination Databases (July 1, 2014)^d^	3184	2015:1426	Not available	Not available	Police and forensic professionals required by law
				2016:1706			
				2017:1904			
				2018:2119			
				2019:2182			
				2020:2469			
				2021:2639			
				2022:2934			
				2023:3184			
				2024: no data available			
*Germany*	2015	From 2015 the German Data Protection Law in conjunction with the German Law for civil servants. Since 2018 § 24 of the BKA Act (Act on the Bundeskriminalamt and the Cooperation between the Federation and the Länder in Criminal Police Matters)^e^	∼2600	2015: 251	194	2021: 93	Employees of the BKA and German Federal Police, as well as visitors with access to forensic areas
				2016: 113			
				2017: 47		2022: 61	
				2018: 77			
				2019: 112		2023: 40	
				2020: 226			
				2021: 460			
				2022: 706			
				2023: 565			
				2024: no data available			
*Netherlands*	2002	No legal framework; agreements with the police and Netherlands Forensic Institute (NFI)	4912	Not provided	Not provided	Not provided	Police staff and NFI research department
*Finland*	March 30, 2007	616/2019 Act on the Processing of Personal Data by the Police^f^	2794	Not available	64	2020: 5	Laboratory and police personnel, expanded over time
						2021: 8	
						2022: 19	
						2023: 32	
						2024: no data available	
*United Kingdom*	2000	Police and Criminal Evidence Act 1984 (PACE)	Not provided	Not provided	2811 contamination events identified by April 2023	2145 crime stain profiles deleted from National DNA database (as of April 2023)	High-risk personnel (e.g., crime scene officers, forensic practitioners); non-police personnel in critical roles (e.g., paramedics, pathologists)

a Pokyn policejního prezidenta č. 275/2016, o identifikačních úkonech”.

b Ustawa o Policji (Dz.U. 1990 nr 30 poz. 179) art. 20 1l, art. 21a-e; Rozporządzenie Ministra Spraw Wewnętrznych (Dz.U. 2020 poz. 1347).

c Until 2024 February.

d Lag (2014:400) om Polismyndighetens elimineringsdatabas

e Bundesdatenschutzgesetz (2015) and Gesetz über das Bundeskriminalamt und die Zusammenarbeit des Bundes und der Länder in kriminalpolizeilichen Angelegenheiten (Bundeskriminalamtgesetz – BKAG) (2018).

f Laki poliisin henkilötietojen käsittelystä (616/2019)0000. 00. 00. 0:00:00.

To contextualize contamination management across jurisdictions, we calculated contamination detection rates where data permitted. These rates, expressed as the number of contamination cases per 1000 elimination DNA profiles, offer a standardized measure for cross-country comparison. In Poland, 403 contamination cases were reported against a total of 9028 elimination samples, resulting in a rate of 44.62 per 1000. In contrast, Czechia reported 1235 contamination cases from 3900 profiles, yielding a substantially higher rate of 316.67 per 1,000, suggesting either greater detection sensitivity or differing procedural standards. Finland documented 64 contamination cases with a total of 2794 profiles, resulting in a detection rate of 22.91 per 1000. Germany reported 194 contamination cases with approximately 2600 profiles, corresponding to a rate of 74.62 per 1000. In the United Kingdom, 2811 contamination events were reported by April 2023, and 2145 crime stain profiles were removed from the National DNA Database. However, the absence of elimination database size data precludes standardized rate calculation. Despite this, the high number of identified and removed profiles indicates a strong contamination response protocol. For Sweden and the Netherlands, either contamination case data or total sample numbers were unavailable or incomplete, making rate calculation infeasible. These disparities across jurisdictions may reflect varying levels of contamination prevention, differences in detection technologies, reporting obligations, or cultural and legal interpretations of what constitutes a reportable event. Without access to standardized protocols, caution is advised when interpreting these rates as indicators of forensic quality or efficiency.

## 4 Discussion

The comparative analysis of data from seven countries reveals significant variability in the establishment, legal frameworks, management, and effectiveness of these databases. These differences underscore both the possibilities and the challenges associated with implementing forensic DNA elimination databases as a standard tool for contamination management.

The results emphasize the critical role of forensic DNA elimination databases in mitigating contamination risks. By storing DNA profiles from personnel with legitimate access to crime scene material, these databases allow investigators to distinguish between real crime scene evidence and contaminant DNA. However, the variability in database size, coverage, and operational protocols across countries suggests the need for greater standardization. For example, countries like Poland and Sweden have developed comprehensive databases with large sample sizes, reflecting robust national frameworks and policies. Poland's database, introduced in 2020, has already amassed over 9000 DNA profiles, while Sweden's database, established in 2014, includes more than 3100 profiles.

The study also illustrates the impact of legal frameworks on the establishment and management of elimination databases. Countries with well-defined legal mandates, such as Finland and Germany, benefit from structured and regulated database operations. In the majority of countries, legally binding documents provide clear guidelines for their use and management. In contrast, the Netherlands lacks a formal legal framework, relying instead on agreements between stakeholders. Despite this regulatory gap, the system is maintained through internal guidelines and institutional best practices. However, the lack of a binding legal mandate may have implications for long-term consistency, oversight, and transparency. For example, no official contamination case data or national reporting metrics are publicly available, making it difficult to assess the effectiveness or comprehensiveness of the Dutch system. This regulatory ambiguity may limit both domestic accountability and international comparability.

The variability in the reported number of contamination cases highlights the importance of elimination databases in maintaining the integrity of forensic investigations. However, the example of France and Hungary, which face legal or institutional barriers to sharing data from their elimination databases, also demonstrates the limitations of their practical utility. In the case of France, data sharing was not possible due to strict legal constraints outlined in the national regulation governing the ‘ORCA’ (outils de recherche de contamination ADN) system. According to Decree No. 2013–406 (from 16th of May 2013), which establishes the rules for DNA contamination traceability tools, personal data processed in the ORCA system may not be disclosed externally. Access to this information is strictly limited to designated personnel within forensic laboratories, and all queries are logged and retained for audit purposes. These legal and procedural safeguards, while crucial for privacy protection, significantly limit the availability of detailed operational data for international research or comparative analysis. Hungary, by contrast, confirmed the existence of a forensic DNA elimination database but declined to provide numerical data, stating only that ‘it is not in their capacity’ to share such information. Unlike France, where legal provisions explicitly restrict data dissemination from the DNA elimination database system, no such legal barriers exist in Hungary. The refusal thus appears to be an institutional decision rather than one mandated by law. This highlights a further challenge in cross-national forensic research: beyond legal obstacles, voluntary institutional non-disclosure may also hinder transparency and scientific collaboration.

These examples underline the pressing need for clear European-level guidance on data sharing within forensic cooperation. The lack of transparency in certain jurisdictions may stem from data protection legislation or institutional hesitancy, both of which may hinder effective cross-border collaboration in forensic science. Developing ethically sound and GDPR-compliant frameworks for intra-European forensic data exchange is essential to overcome these limitations.

The research findings clearly highlight the importance of establishing and effectively utilizing forensic DNA elimination databases to ensure the reliability and integrity of investigations. In the authors' view, the variability in practices and regulations identified in this study underscores the need for harmonized frameworks at the European level. Standardized guidelines for database establishment, management, and data sharing could enhance the effectiveness of forensic DNA elimination databases and promote cross-border collaboration. Additionally, integrating best practices from countries with successful databases, such as Czech, Poland, and Sweden, could serve as a model for other nations. For example, Poland's rapid implementation and large-scale database coverage could serve as a model for efficient expansion, while Sweden's legal framework and steady database growth demonstrate the importance of regulatory clarity and long-term planning. Czechia's comprehensive approach to documenting contamination cases highlights the value of detailed reporting protocols for improving database utility.

The comparative findings indicate that countries such as Czechia, Poland, and Sweden employ robust elimination database systems that may serve as models for emerging or less regulated systems**.** Czechia's legally binding framework, coupled with detailed annual reporting, enables traceability and transparency. Poland demonstrates how rapid national implementation can yield broad coverage in a short period, while Sweden's example underscores the value of clear statutory foundations. Countries like the Netherlands, which operate databases without explicit legal mandates, may benefit from transitioning toward formal regulatory structures to enhance sustainability and accountability. Policymakers may consider adopting standardized contamination reporting metrics, such as ‘cases per 1000 profiles’, to improve comparability across Europe.

### 4.1 Future directions

Effective contamination management remains a cornerstone of forensic DNA reliability and requires a combination of robust operational protocols, advanced technologies, and continuous training of personnel. Countries with well-established elimination databases exemplify the importance of detailed reporting mechanisms and systematic comparisons of DNA profiles. Including high-risk personnel, such as crime scene investigators and laboratory staff, is also critical to minimizing contamination risks. Future advancements in this area should focus on developing automated detection systems and integrating anonymized international elimination databases to enhance cross-border investigations and collaboration.

Harmonization at the European level is not only necessary to improve database practices but also to build public trust by demonstrating a unified approach to balancing operational efficiency with respect for fundamental rights. Transparent communication about the purpose, limitations, and benefits of these databases—alongside strict compliance with ethical and legal standards—will be vital in fostering acceptance across jurisdictions. Addressing these challenges in future research and policy discussions will contribute to the development of effective, trustworthy, and harmonized forensic DNA elimination practices across Europe.

Given the critical importance of the ethical, legal, and social implications (ELSI) of forensic DNA elimination databases in shaping harmonized European frameworks, future research should focus on these dimensions. Key areas to explore include privacy concerns, data protection measures, and the societal impacts of such databases. By integrating ELSI considerations into policy development, countries can ensure that forensic DNA elimination databases respect individual rights while effectively fulfilling their operational goals.

Based on the comparative analysis, we recommend the following:

• Adoption of standardized contamination rate metrics across jurisdictions to enable meaningful benchmarking.
• Introduction of legally binding regulatory frameworks in countries operating on informal agreements.
• Publicly available annual reporting to ensure transparency and foster trust in forensic institutions

### 4.2 Study limitations

The study's limitations include the response rate, as not all ENFSI member states' forensic DNA laboratories based in Europe provided data. Future research should prioritize engaging these non-responding countries to develop a more complete and representative understanding of forensic DNA practices across Europe. Expanding the dataset would enable a more detailed analysis of regional variations and facilitate the identification of best practices.

It is important to note that detailed national protocols and operational procedures regarding contamination detection and reporting were not available to the authors. Consequently, the differences in the number of reported contamination cases across countries cannot be conclusively attributed to better contamination control, higher sensitivity in detection, or mere procedural variance. These figures may reflect varying levels of transparency, legal requirements for reporting, or even differences in case definitions. Therefore, while numerical comparisons are informative, caution must be exercised in interpreting them as indicators of actual forensic laboratory performance.x.

## 5 Conclusion

Forensic DNA elimination databases play an essential role in safeguarding the reliability of DNA evidence by minimizing the risks associated with contamination. This study highlights significant variability in the implementation, management, and legal frameworks of these databases across Europe, reflecting diverse operational and regulatory contexts. Countries such as Poland, Sweden, Czechia, and the UK exemplify robust frameworks and practices, offering valuable models for others to emulate. Conversely, barriers in data sharing and legal provisions, as observed in France, along with a lack of willingness to share data without explanation in Hungary, underscore the pressing need for harmonized European frameworks.

Standardized guidelines that address the establishment, management, and ethical use of forensic DNA elimination databases are crucial for fostering transparency, cross-border collaboration, and public trust. By incorporating ethical, legal, and social considerations into future policy development, European countries can ensure that forensic DNA elimination databases not only achieve their intended objectives but also uphold the fundamental rights of individuals. This harmonization effort will strengthen forensic science and criminal justice systems, contributing to more reliable and effective investigations across the continent.

## CRediT authorship contribution statement

**Mónika Nogel:** Writing – original draft, Visualization, Formal analysis, Data curation, Conceptualization. **Gábor Kovács:** Writing – original draft. **Zsolt Pádár:** Writing – original draft.

## Funding

This research did not receive any specific grant from funding agencies in the public, commercial, or not-for-profit sectors.

## Declaration of competing interest

The authors declare that they have no known competing financial interests or personal relationships that could have appeared to influence the work reported in this paper.
